# Evolution of within-colony distribution patterns of birds in response to habitat structure

**DOI:** 10.1007/s00265-014-1697-8

**Published:** 2014-03-04

**Authors:** Piotr Minias

**Affiliations:** Department of Teacher Training and Biodiversity Studies, University of Łódź, Banacha 1/3, 90-237 Łódź, Poland

**Keywords:** Birds, Ciconiiformes, Coloniality, Comparative analysis, Distribution, Habitat structure

## Abstract

It has long been suggested that habitat structure affects how colonial birds are distributed within their nesting aggregations, but this hypothesis has never been formally tested. The aim of this study was to test for a correlated evolution between habitat heterogeneity and within-colony distributions of Ciconiiformes by using Pagel’s general method of comparative analysis for discrete variables. The analysis indicated that central-periphery gradients of distribution (high-quality individuals occupying central nesting locations) prevail in species breeding in homogeneous habitats. These were mainly ground-nesting larids and spheniscids, where clear central-periphery patterns were recorded in *ca*. 80 % of the taxa. Since homogeneous habitats provide little variation in the physical quality of nest sites, central nesting locations should be largely preferred because they give better protection against predators by means of more efficient predator detection and deterrence. By contrast, central-periphery gradients tended to be disrupted in heterogeneous habitats, where 75 % of colonial Ciconiiform species showed uniform patterns of distribution. Under this model of distribution, edge nest sites of high physical quality confer higher fitness benefits in comparison to low-quality central sites, and thus, high-quality pairs are likely to choose nest sites irrespectively of their within-colony location. Breeding in homogeneous habitats and uniform distribution patterns were identified as probable ancestral states in Ciconiiformes, but there was a significant transition rate from uniform to central-periphery distributions in species occupying homogeneous habitats.

## Introduction

According to classic theoretical predictions on resource acquisition in animals, individuals should be distributed in environments so as to maximise their fitness. If there are no competitive asymmetries between conspecifics, the habitat should be occupied proportionally to the available resources under the assumptions of the ideal free distribution model of Fretwell and Lucas (1970), which implies that the density of individuals should increase along with the increasing quality of the habitat patch. However, in natural populations of animals, individuals only rarely gain equal access to resources, as they notably differ in their competitive abilities. Conforming to the empirical evidence on competitive asymmetries within populations, a model of ideal despotic distribution was proposed, assuming that dominant individuals have capacities to secure the best available territories and to relegate conspecifics of lower phenotypic quality to less attractive habitats (Fretwell 1972).

Although there is abundant empirical evidence for ideal despotic distributions in territorial birds (Andrén 1990; Ens et al. 1995; Møller 1995; Petit and Petit 1996), information on the patterns of distribution in colonial species are much more scarce. Distribution patterns of colonial birds are typically considered on at least three different levels: (1) distribution of colonies in the available environment; (2) distribution of individuals among colonies; and (3) distribution of individuals within colonies. Large-scale patterns of colony distribution as well as distribution of individuals among the colonies located in the habitat patches of different quality have both recently received increasing attention (Brown and Rannala 1995). It has been demonstrated that the foundation of new colonies in several avian species follows a despotic model (Serrano and Tella 2007; Oro 2008) and that individuals of low phenotypic quality (expressed by young age or poor physical quality) may be excluded from colonies that are located in the best habitat patches (Rendón et al. 2001).

By contrast, much less empirical data has been collected on the distribution of individuals within their breeding colonies. Despite this relative scarcity of information, it seems safe to distinguish two major components of nesting site attractiveness that may have important fitness consequences for colonially breeding species and that, consequently, are likely to determine within-colony distribution patterns. The first of the components is associated with the within-colony location of nest sites, as it is generally accepted that the centres of colonies offer the highest benefits in terms of fitness (Coulson 1968; Aebischer and Coulson 1990). It has been commonly reported that pairs nesting in the centres are likely to achieve higher breeding success due to decreased predation-related losses of eggs and chicks (Götmark and Andersson 1984; Yorio and Quintana 1997; Minias and Kaczmarek 2013), thus indicating that colonies may act as selfish herds against predation (Brown and Brown 2001). The mechanisms explaining lower susceptibility of central pairs to predation may include more efficient detection and deterrence of predators in the central parts of colonies. Central nests are also likely to be less accessible to predators, although this may largely depend on the type of predator (Brunton 1997). However, in general, there is large empirical support that colonial birds dilute the risk of predation (Brown and Brown 2001) and that central nesting sites are by far the most efficiently protected sites against predators. Although the breeding success of centrally nesting pairs may be decreased due to density-dependent intraspecific interactions (Jovani and Grimm 2008; Ashbrook et al. 2010) and parasitic rates (Tella 2002), many studies have demonstrated higher reproductive output in colony centres in comparison to the edges (Patterson 1965; Gochfeld 1980; Becker 1995; Vergara and Aguirre 2006). For this reason individuals of higher quality are likely to occupy the best central sites and to relegate individuals of lower quality to less attractive edge sites. Assuming such a despotic mechanism of colony formation, one would expect a central-periphery pattern of distribution where the phenotypic quality of breeding birds declines from the centre towards the edges of a colony (Coulson 1968).

The physical quality of nesting sites may be considered as the second major component of their attractiveness for colonial birds. If the habitat is heterogeneous on a small spatial scale, considerable variation in the physical quality of nesting sites within colonies is expected. Under this assumption, nest sites of high quality would be likely to provide much more effective protection against predators or adverse weather conditions and thus would promote higher reproductive success. It has been suggested that if the fitness benefits of nesting in sites of good physical quality considerably exceeds benefits associated with the central nesting position, then high-quality pairs may choose the best available nesting sites independently of their location in a colony (Velando and Freire 2001). Under such circumstances, the central-periphery patterns could be disrupted and pairs of varying quality could be distributed more or less uniformly among the central and peripheral zones of colonies.

Although the effects of habitat heterogeneity on the within-colony patterns of distribution in birds have long been hypothesised, most of the empirical evidence has only been circumstantial and the suggested relationship has never been supported by a formal analysis. The aim of this study was to test for evolutionary correlations between the structure of breeding habitat and within-colony distributions in Ciconiiformes (sensu Sibley and Ahlquist 1990), a phylogenetic group with the highest prevalence of coloniality among birds (Siegel-Causey and Kharitonov 1990). According to the theoretical predictions, I expected that birds nesting in homogeneous habitats should form colonies according to the central-periphery model of distribution, whereas in heterogeneous habitats, the central-periphery gradients should be disrupted (uniform model of distribution).

## Methods

For the purpose of the analysis, I collected data from the literature for 34 colonial species of Ciconiiformes grouped into nine families (Table 1). Each species was assigned a prevailing model of within-colony distribution: central-periphery or uniform. The central-periphery model was assigned when all studied reproductive parameters or parental quality traits declined from the centre of the colony towards the peripheries. In contrast, the uniform model corresponded to a situation in which the central-periphery gradients were disrupted, at least with respect to some of the studied traits or in some of the studied colonies. With such an approach, the distribution patterns could be coded binarily, with central-periphery distributions denoted as 0 and distributions in which central-periphery gradients were disrupted (uniform models) denoted as 1. Heterogeneity of the breeding habitat was also treated as a categorical variable with two states. Bare ground and mats of floating vegetation were identified as homogeneous habitats, as these provide none or negligible variation in the physical quality of nesting sites and all nests are more or less equally vulnerable to predators or adverse weather conditions (denoted as 0). All of the other habitats that may provide moderate or considerable variation in the physical quality of nesting sites were considered to be heterogeneous habitats (denoted as 1). This category mostly included rocky habitats (cliff ledges, rocky slopes and islets, rock crevices, hollows and burrows) and vegetated habitats (woodlands and shrubs).

**Table 1:** Patterns of within-colony distribution and nesting habitat of colonial Ciconiiform species

Species	Distribution	Habitat	Parameters	Authors
Spheniscidae				
*Aptenodytes patagonicus*	U	Ground	BD (C-P)	Côté 2000
			BD, RS (C-P)	Bried and Jouventin 2001
			AS, RS (U)	Decamps et al. 2009
*Eudyptes chrysocome*	C-P	Ground	RS	Hull et al. 2004
*Spheniscus magellanicus*	C-P	Ground	CS, S, RS	Gochfeld 1980
			S, RS	Frere et al. 1992
*Pygoscelis antarcticus*	C-P	Ground	AM	Mínguez et al. 2001
			BD	Barbosa et al. 1997
*Pygoscelis adeliae*	C-P	Ground	RS	Taylor 1962
			CS, RS	Tenaza 1971
			BD, CS	Spurr 1975
			CS, S	Davis and McCaffrey 1986
Procellaridae				
*Thalassarche melanophris*	C-P	Ground	RS	Forster and Phillips 2009
Pelecanidae				
*Pelecanus occidentalis*	C-P	Ground	AA, CS, RS	Blus and Keahey 1978
Ciconiidae				
*Ciconia ciconia*	C-P	Trees	AA, RS	Vergara and Aguirre 2006
Ardeidae				
*Nycticorax nycticorax*	U	Trees	CS (U); RS (C-P)	Uzun 2009
*Bubulcus ibis*	U	Trees	S (C-P)	Siegfried 1972
			HS (U)	Ranglack et al. 1991
			CS (U)	Samraoui et al. 2007
*Ardea cinerea*	U	Trees	RS	Van Vessem and Draulans 1986
*Egretta garzetta*	U	Trees	CS (U); RS (C-P)	Uzun and Kopij 2010
Phalacrocoracidae				
*Phalacrocorax atriceps*	U	Rocks	AA	Shaw 1985
			RS	Svagelj and Quintana 2011
*Phalacrocorax aristotelis*	U	Rocks	RS	Velando and Freire 2001
*Phalacrocorax pelagicus*	U	Cliff	BD, AA	Siegel-Causey and Hunt 1986
*Phalacrocorax carbo*	U	Trees	RS (U)	Grieco 1994
			BD (C-P)	Andrews and Day 1999
			BD, RS (C-P); CS (U)	Minias et al. 2012a
			BD, CC, RS, S (C-P); CS (U)	Minias and Kaczmarek 2013
*Phalacrocorax auritus*	C-P	Trees	BD	Léger and McNeil 1987
Sulidae				
*Morus capensis*	C-P	Ground	AA, RS	Staverees et al. 2008
*Morus serrator*	U	Ground	BD, AA (C-P)	Gibbs et al. 2000
			AA (U)	Pyk et al. 2008
*Sula variegata*	U	Rocks	BD	Duffy 1983
*Sula leucogaster*	U	Rocks	RS	Ospina-Alvarez 2008
Accipitridae				
*Pandion haliaetus*	C-P	Trees	RS	Hagan and Walters 1990
Laridae				
*Hydroprogne caspia*	C-P	Ground	BD, RS	Antolos et al. 2006
*Sternula antillarum*	U	Ground	HS, S, RS	Brunton 1997
*Thalasseus maximus*	C-P	Ground	BD, S	Buckley and Buckley 1977
*Chlidonias hybridus*	C-P	Floating vegetation	CS	Minias et al. 2011
			CGR	Minias et al. 2012b
			S	Minias et al. 2013
*Sterna dougallii*	C-P	Ground	BD	Ramos 2002
*Sterna hirundo*	C-P	Ground	BD, S, RS	Becker 1995
*Chroicocephalus ridibundus*	C-P	Ground	RS	Patterson 1965
*Rissa tridactyla*	U	Cliff	AS (C-P)	Coulson and Wooller 1976
			RS (U)	Wooller and Coulson 1977
			S (C-P); RS (U)	Regehr et al. 1998
			AS (C-P)	Aebischer and Coulson 1990
*Larus atricilla*	C-P	Ground	BD, CS, S, RS	Montevecchi 1978
*Larus delawarensis*	U	Ground	AA (C-P)	Ludwig 1974
			HS, S, RS (C-P)	Dexheimer and Southern 1974
			AA (C-P)	Ryder 1975
			BD, CS, HS, RS (U)	Ryder and Ryder 1981
			BD, AA (C-P)	Haymes and Blokpoel 1980
*Larus californicus*	C-P	Ground	RS, AA	Pugasek and Diem 1983
*Larus argentatus*	C-P	Ground	BD	Burger and Shisler 1980

Central-periphery (*C-P*) and uniform (*U*) patterns were assigned to within-colony distributions of the following traits: *BD* breeding date, *CS* clutch size, *HS* hatching success, *RS* reproductive success, *S* brood survival, *CGR* chick growth rates, *CC* chick condition, *AS* adult survival, *AA* adult age, *AM* adult morphology. If different, the patterns of distribution were indicated in parentheses separately for each reported reproductive/quality trait or for each studied colony

**Fig. 1:**
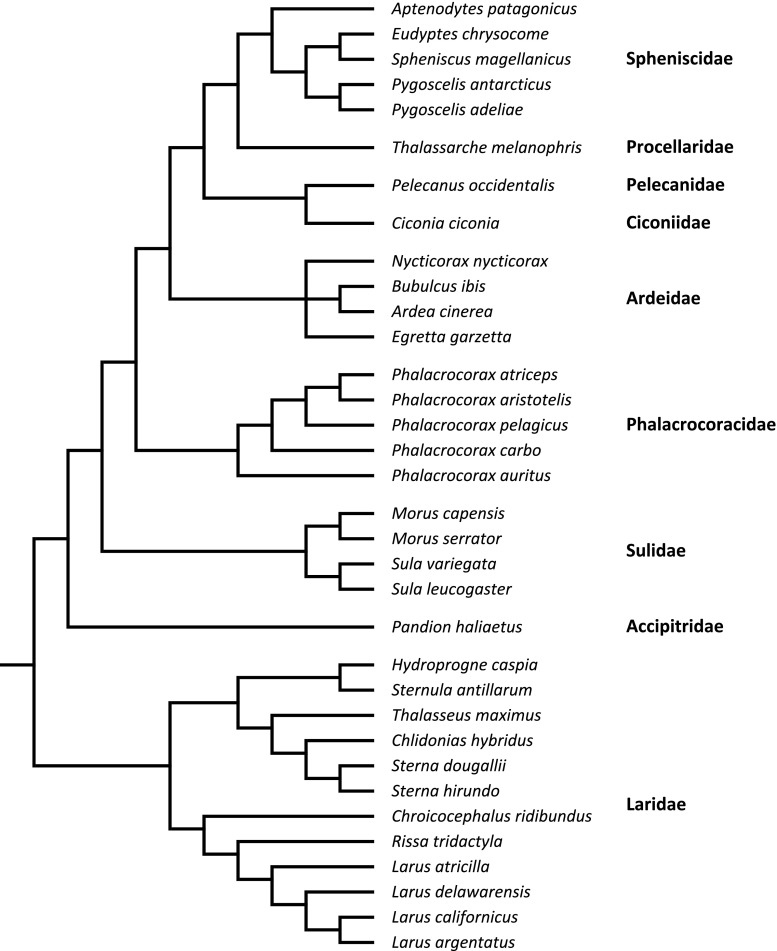
Phylogenetic tree of 34 colonial species from nine Ciconiiformes families involved in the study

Since data from different species are not independent due to their shared ancestral states, it is widely acknowledged that comparative analyses must control for the phylogeny. I based my phylogenetic tree on the classification of Sibley and Ahlquist (1990). This phylogeny is uniquely available for the entire order of Ciconiiformes, and for this reason, it was used to branch the families of my tree (Fig. 1). Although the phylogeny of Sibley and Ahlquist (1990) was once considered controversial (Sheldon and Gill 1996), it is now assumed as quite robust for phylogenetic analyses in birds (reviewed in Mooers and Cotgreave 1994), and hence, it has been broadly used in comparative studies (Cézilly et al. 2000; Dubois and Cézilly 2002; Garamszegi et al. 2005; Olson et al. 2008), including those on avian coloniality (Rolland et al. 1998; Varela et al. 2007). In order to branch the genera and species, I used five phylogenies based on both molecular and morphological data (Sheldon 1987; Kennedy et al. 2000; Thomas et al. 2004; Bertelli and Giannini 2005; Smith 2010). Since different approaches were used to construct the above phylogenies, I decided not to control for branch lengths, which followed from other comparative studies (Dubois et al. 1998; Rolland et al. 1998; Cézilly et al. 2000; Varela et al. 2007). Setting equal branch lengths is considered conservative (Pagel 1994), and it has been demonstrated that it does not bias the results qualitatively (Møller et al. 1998; Nunn 1999).

To test for a correlated evolution between breeding habitat structure and within-colony distribution patterns, I used Pagel’s discrete variable method (1994) which uses the continuous-time Markov model in order to characterise evolutionary changes in selected pairs of variables along each branch of the phylogenetic tree. The method compares the fit of two different models assuming an either independent or dependent (the rate of change of one trait depends on the background state of the other) evolution of traits. The models were fitted using maximum likelihood and compared using the likelihood ratio (LR) statistic, which is expressed as LR = 2 (log*L*_(D)_ − log*L*_(I)_), where *L*_(D)_ is the likelihood of the model that allows the traits to evolve in a correlated fashion and *L*_(I)_ is the likelihood of the independent model. The LR statistic is asymptotically distributed as *χ* ^2^ with four degrees of freedom for this test (Pagel 1997).

The discrete variables method was also used to estimate the ordering and direction of the evolutionary changes of the two analysed variables (so-called temporal order tests). For this purpose, one needs to fit reduced models in which a certain rate of evolutionary transition *q*_*ij*_ is excluded a priori (set to 0). The constrained seven-parameter models are then compared to the full eight-parameter model which tests the hypotheses whether the specified transition rates differ significantly from zero. The tests are asymptotically distributed as *χ* ^2^ with one degree of freedom (Pagel 1994). In this manner, the evolution between the ancestral states and the derived states of both selected variables may be traced. For root reconstruction of ancestral states, I used the maximum-likelihood reconstruction method of Pagel (1999). All analyses were performed with BayesTraits (Pagel and Meade 2008).

## Results

**Fig. 2:**
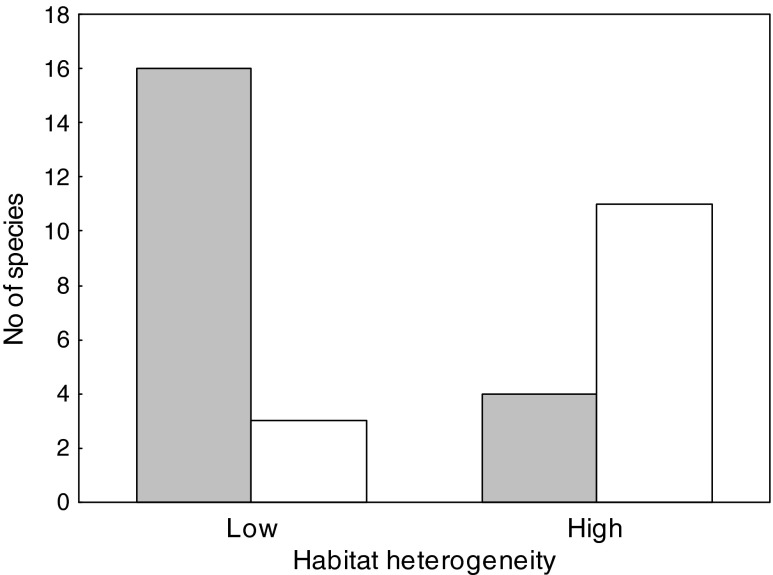
Number of species exhibiting central-periphery (*grey area*) and uniform (*white area*) patterns of within-colony distribution with respect to the heterogeneity of breeding habitat

Among the 34 taxa used for the analysis, there were 20 species that nested in homogeneous habitats (59 %) and 14 that chose heterogeneous habitats for breeding (41 %). A similar proportion was found between the number of species that exhibited central-periphery distribution within colonies and those in which central-periphery gradients were disrupted (56 vs. 44 %, Table 1). There was a clear tendency for species breeding in homogeneous habitats to be distributed central-peripherally within the colonies (Fig. 2). The log-likelihood of the model of independent evolution was estimated at *L*_0_ = −36.10 and was significantly lower in comparison to the likelihood of the dependent model *L*_1_ = −30.18 (*χ* ^2^ = 11.83, df = 4, *P* = 0.019). Such results support the hypothesis of a correlated evolution between preferences for heterogeneous breeding habitats and uniform patterns of within-colony distribution.

Breeding in homogeneous habitats and uniform distribution of pairs within colonies were identified as ancestral states with a probability of 94.8 %. I found a significant rate of transition from uniform to central-periphery distribution in species that bred in homogeneous habitats (*χ* ^2^ = 4.54, df = 1, *P* = 0.033). I also found a significant rate of reversed transitions, i.e. from central-periphery to uniform patterns of distribution (*χ* ^2^ = 7.50, df = 1, *P* = 0.006). The rate of evolution from breeding in homogeneous to heterogeneous habitats with no change in the background state of uniform distribution pattern was not significant (*χ* ^2^ = 1.14, df = 1, *P* = 0.29), but it cannot be excluded that this could have resulted from the low power of the test. All the other transition rates were also non-significant (all *P* > 0.05).

## Discussion

This study was the first to formally demonstrate a link between within-colony distribution patterns in birds and the structure of preferred nesting habitat by using comparative analysis. It was shown that as much as 85 % of colonial Ciconiiformes species which breed in homogeneous habitats tend to show clear central-periphery patterns of distribution within their colonies and that these are mostly ground-nesting species from the Spheniscidae and Laridae families. In general, bare-ground habitats, such as sandy islands or dunes, provide no apparent variation in the physical quality of the nesting sites. Under such conditions, each nest site is likely to be equally exposed to predation and inclement weather. Consequently, nest-site selection patterns should evolve towards choosing an appropriate location within the colony, where pressure coming from predators will be minimised. Assuming that all nest sites are physically similar, the highest fitness benefits are expected to be acquired via nesting in the central parts of colonies (Coulson 1968). As colony centres are usually associated with higher nesting densities, the mechanisms which may explain lower predation rates at these locations include: (1) restricted accessibility for predators (Siegel-Causey and Hunt 1981); (2) more efficient communal defence (Elliot 1985); (3) more efficient detection of predators (Roberts 1996); and (4) lower probability of being depredated due to the dilution effect (Murphy and Schauer 1996).

By contrast, heterogeneous habitats were found to disrupt the central-periphery patterns of distribution within colonies. In habitats of moderate or high heterogeneity, edge nest sites of high physical quality are likely to confer higher fitness benefits in comparison to low-quality central sites. Thus, high-quality pairs are expected to choose nest sites irrespectively of their within-colony location, and thus, they are expected to be uniformly distributed among the central and peripheral zones of colonies. Central-periphery distributions were found to be disrupted in nearly 75 % of colonial Ciconiiformes species that nested in heterogeneous habitats. It seems that uniform patterns of distribution are especially common in birds that establish colonies on cliffs or in other rocky habitats, including various Phalacrocoracidae and Sulidae species. Nesting sites such as crevices under fallen rocks, open ground caves and open ledges on cliffs usually show great variation in their physical quality and attractiveness for birds; for example, a clear preference for sites with more lateral and overhead cover, with better drainage and with better visibility has been demonstrated for the European Shag *Phalacrocorax aristotelis* (Velando and Freire 2003). Such physical characteristics of nesting sites have been shown to provide more effective protection against predators and to prevent broods from flooding, unfavourable atmospheric conditions and intra-specific inference, which greatly affected the hatching success of Shags (Velando and Freire 2003). The distribution of birds within the same colony of Shags did not conform to the assumptions of the central-periphery model, as individuals of different quality were distributed despotically among the sites of varying physical quality (Velando and Freire 2001, 2003).

Disruptions in the central-periphery patterns of distribution were also recorded in several waterbird species associated with woodland habitats; although, this kind of environment is expected to provide only moderate variation in the physical quality of nesting sites, most commonly expressed by variation in tree height and canopy structure. In several tree-nesting colonial avian species, tree height was identified as an important predictor of reproductive success and was suggested to determine accessibility of nests to ground and tree-dwelling predators (Post 1990; Childress and Bennun 2000). The breeding success of the Scarlet Ibis *Eudocimus ruber* correlated positively with nest cover by overhanging branches (Olmos 2003), and the study on Cattle Egrets *Bubulcus ibis* indicated higher fledging success in pairs nesting close to the trunks of trees (Si Bachir et al. 2008). However, in some cases, the fitness benefits that were associated with nesting in the sites of high physical quality could be acquired via mechanisms not related to anti-predatory protection; for example, in the tree-nesting subspecies of Great Cormorant *Phalacrocorax carbo sinensis*, the physical quality of nesting sites (tree height) determined the probability of nest collapse before the conclusion of breeding activities (Minias and Kaczmarek 2013).
